# Expert opinion of an Italian working group on the assessment of cognitive, psychological, and neurological outcomes in pediatric, adolescent, and adult patients with phenylketonuria

**DOI:** 10.1186/s13023-022-02488-2

**Published:** 2022-12-21

**Authors:** Filippo Manti, Stefania Caviglia, Chiara Cazzorla, Annamaria Dicintio, Andrea Pilotto, Alessandro P. Burlina

**Affiliations:** 1 Unit of Child Neurology and Psychiatry, Department of Human Neuroscience, University of Rome La Sapienza, Rome, Italy; 2 Unit of Clinical Psychology, Department of Neurosciences, Bambino Gesù Children’s Hospital, IRCCS, Rome, Italy; 3 Division of Inborn Metabolic Diseases, Department of Pediatrics, University Hospital, Padua, Italy; 4 Department of Metabolic Diseases and Clinical Genetics, Giovanni XXIII Children Hospital, Azienda Ospedaliero-Universitaria Consorziale Policlinico, 70126 Bari, Italy; 5 Neurology Unit, Department of Clinical and Experimental Sciences, University of Brescia, Brescia, Italy; 6 Neurology Unit, St. Bassiano Hospital, Bassano del Grappa, Italy

**Keywords:** Phenylketonuria, Quality of life, Follow-up, Psychology, Neurology, Brain magnetic resonance imaging, Expert opinion

## Abstract

Phenylketonuria (PKU) is an inherited metabolic disease characterized by a defective conversion of phenylalanine (Phe) to tyrosine, potentially leading to Phe accumulation in the brain. Dietary restriction since birth has led to normal cognitive development. However, PKU patients can still develop cognitive or behavioral abnormalities and subtle neurological deficits. Despite the increasing evidence in the field, the assessment of neurocognitive, psychopathological, and neurological follow-up of PKU patients at different ages is still debated. The high interindividual variability in the cognitive outcome of PKU patients makes the specificity of the neurocognitive and behavioral assessment extremely challenging. In the present paper, a multidisciplinary panel of Italian PKU experts discussed different tools available for cognitive, psychopathological, and neurological assessment at different ages based on the existing literature and daily clinical practice. This study aims to provide evidence and a real-life-based framework for a specific clinical assessment of pediatric, adolescent, and adult patients affected by PKU.

## Background

Phenylketonuria (PKU) is a rare inherited metabolic disorder (IMD) caused by a deficiency in the phenylalanine (Phe) hydroxylase (PAH) enzyme, impairing the conversion of the amino acid Phe to tyrosine. The incidence of PKU in Europe is around 1/10,000–1/15,000 births, but it is higher in some countries, including Italy, where it reaches 1/4500 [1]. Deficiency of the hepatic PAH leads to a broad spectrum of hyperphenylalaninemia (HPA). HPAs are classified according to the treatment options: non-PKU HPA (Phe concentration ranging from 120 to 360 μmol/L) and PKU HPA (blood Phe concentration > 360 μmol/L) [2, 3]. The consequent accumulation of Phe to toxic concentrations in the brain results in severe clinical, neuropsychological, neurophysiologic, biochemical, and imaging alterations in untreated patients [4–7]. In particular, high Phe levels (> 600 μmol/L) could be associated with different neurotransmitters deficits and white matter alterations [8]. Thanks to the neonatal screening for PKU, patients can be treated in their early days while growing up, thus avoiding severe neurological deficits. However, a relevant percentage of patients treated early in their childhood still exhibit subtle cognitive deficits and psychosocial alterations in adulthood [9, 10]. In PKU patients treated early, prolonged high levels of Phe, particularly in adolescence, could negatively impact the individual’s cognitive functions [7, 11]. Unfortunately, nowadays, the chance of a pre-screening patient with a severe cognitive deficit arriving at the center is still possible.

There is a large consensus about the importance of analyzing the impact of neurocognitive deficits in PKU patients; however, it seems difficult to define a standardized and systematic neurocognitive patient assessment, as this strictly depends on age and severity of deficits [12].

The first-line treatment of PKU is based on a low Phe diet in combination with a protein substitute (mixtures of amino acids Phefree). Adherence to diet commonly decreases from childhood to adulthood; this event should be avoided, as hyperphenylalaninemia also impacts the adult brain [13, 14].

Until 2018, the only pharmacological therapy approved for PKU was the supplementation of tetrahydrobiopterin (BH_4_), an enzymatic co-factor of PAH. The tetrahydrobiopterin drug (Kuvan®) is the oral form of sapropterin dihydrochloride [15]. BH_4_ is not available worldwide, and only a proportion (estimated 30%) of PKU patients can respond to this treatment [16]. In 2018 and 2019, the US Food and Drug Administration (FDA) and the European Medicine Agency (EMA) approved pegvaliase (Palynziq®, BioMarin Pharmaceutical), respectively; pegvaliase is a pegylated recombinant *Anabaena variabilis*-derived Phe ammonia lyase able to reduce blood Phe concentration by substituting for Phe hydroxylase and converting Phe to ammonia and trans-cinnamic acid [17]. With pegvaliase, approved for patients ˃ 16 years old and with Phe levels > 600 μmol/L, patients can follow a diet with an amount of protein intake meeting the recommended dietary intake for the general population and even liberalize their diet [18, 19].

### Outcomes in PKU and available tools for the assessment

#### Cognitive functions

Several single and multicenter studies showed that children, adolescents, and adults with PKU, even if treated from birth, may exhibit deficits in several domains [20–23], mainly executive functions (CFx) and attention [24–27]. CFx are responsible for goal-directed or future-oriented behavior, including initiating activity, impulse control, self-regulation, working memory, mental flexibility, planning, and organization ability [28]. The most consistent impairments of CFx in PKU patients have been observed in working memory, sustained attention, and inhibitory control [10, 29]. However, the degree of deficits and impairments broadly vary among different studies [10, 30]. This may be due to researchers' different ways of conceptualizing CFx and employing various CFx assessment tasks [31].

Several neuropsychological tests are available to assess cognitive performance in PKU patients but finding the best tool for the specific patient and impairment is not trivial, as no standard method for PKU patients exists. On the one hand, intellectual quotient (IQ) evaluation usually provides a reliable assessment of general cognitive functioning; on the other, it is not sensitive enough to detect minor neurocognitive dysfunctions [32]. As neuropsychological and behavioral alterations in PKU are heterogeneous in terms of degree and domains impaired, it is essential to identify the right tool for the right patient (according to the phenotype and age). Moreover, identifying a tool/pool of tools able to assess the overall burden of illness of PKU patients is crucial [33].

The central issue in assessing CFx in PKU populations is the lack of consistent use of valid and sensitive tests suitable for both children and adults [29]. Moreover, many traditional CFx tasks depend on multiple cognitive processes and show significant variability in the results. In the future, it will be essential to define a specific set of neuropsychological tasks to be used across international PKU centers [10, 14].

#### Quality of life, emotional and behavioral symptoms in PKU

PKU is associated with an increased incidence of emotional and behavioral problems [34–36] that could impair the Quality of Life (QoL) of patients [11]. Children and adults with PKU show emotional troubles such as low self-esteem, lower achievement motivation, decreased autonomy and reduced social competence. In contrast, adolescents and adults tend to have mood and anxiety disorders and social isolation [11, 37–39]. Patients with PKU often avoid meeting with friends, traveling, and performing sports, and recreational activities, with a significant impact on their QoL [40]. The management of a PKU patient should include an emotional and behavioral assessment and be aimed at identifying psychiatric disorders to allow for early treatment. The presence of emotional and behavioral troubles in PKU patients is possibly the consequence of growing up and living with a chronic disease [39].

Despite the evidence mentioned above, studies assessing the QoL of PKU patients provided conflicting results; in some cases, the results were similar to those of the general population [41–43]. In others, the studies showed a worsening of this parameter [44, 45]. Future studies, as recommended by European (EU) guidelines [14], should use the recently developed PKU-QoL questionnaire that is specifically designed to detect the impact of PKU on all aspects of a patient's life [46, 47].

#### Neurological outcome

Neurological signs (i.e., tremor, spastic paraparesis, and ataxia) are described in approximately 90% of untreated PKU patients and in a high proportion of late-treated PKU patients [48]. However, also in early treated PKU patients, some neurological signs, such as brisk tendon reflexes, clumsy motor coordination, and tremor, are frequently reported [47–49]. More recently, in a cohort of French adult PKU patients, the incidence of neurological complications has accounted for 5.1% [50].

Neuroimaging studies revealed that early-treated PKU adults exhibit a wide range of brain abnormalities, mostly related to white matter involvement [9]. Most patients exhibit mild to moderate white matter hyperintensities with no cortical atrophy or gray matter lesions [8, 9]. The degree of white matter alterations has been associated with mean Phe levels, but there are contradicting data addressing the relationship between dietary adherence and the severity of brain abnormalities [9]. No studies show a strict correlation between the degree of burden of white matter hyperintensities and the severity of cognitive or behavioral abnormalities, making magnetic resonance imaging (MRI) assessment questionable, except in a research setting. Nevertheless, the paper by Jaulent and colleagues recently showed that in a limited number of patients, who resumed or initiated a low-Phe diet, there was a partial/total regression of white matter abnormalities [50].

Indeed, the conventional brain MRI [(FLAIR/T2-weighted imaging and diffusion-weighted imaging (DWI)] is a powerful, readily available, noninvasive tool for detecting brain changes in PKU patients [9]. In line with this, EU guidelines and the Italian national consensus statement on management and pharmacological treatment of PKU suggest that neuroimaging examinations should be reserved only for those patients presenting with an atypical clinical course and/or unexpected neurological deficits, or for research purposes [3, 14].

### Aim

Available scientific literature highlights the importance of a specific neurocognitive, psychopathological, and neurological assessment in PKU [9].

The different degrees of neurological and behavioral abnormalities of early-treated PKU patients (especially adults) are still debated, and the choice of the “best” neurocognitive test, sensitive to high levels of Phe, remains a challenge. To make things more complicated, a remarkable interindividual variability in the cognitive outcome and an inconsistent correlation between cognitive performances and biochemical control have been observed, suggesting the presence of an individual resilience or vulnerability to Phe in young early-treated adults [51].

Therefore, there is a significant need to reach an agreement on an appropriate set of tests according to the age of patients. The tasks should be suitable for use in everyday clinical practice. To fill this gap, a multidisciplinary group of Italian experts in the PKU field has assembled to propose a more comprehensive neurocognitive, psychopathological, and neurological assessment of PKU patients based on the existing literature and their clinical experience.

## Methods

### The panel of experts

The panelists, also authors of this study, gathered in two meetings. The six panelists work in Italian centers with extensive experience in PKU management and different medical specialties (neurology, neuropsychiatry, psychology, and inherited metabolic diseases). In particular, the panel was constituted of two adult neurologists (Alessandro Burlina and Andrea Pilotto), three psychologists (Stefania Caviglia, Chiara Cazzorla, Annamaria Dicintio), and one child neuropsychiatrist (Filippo Manti).

### Expert opinion

**Fig. 1:**
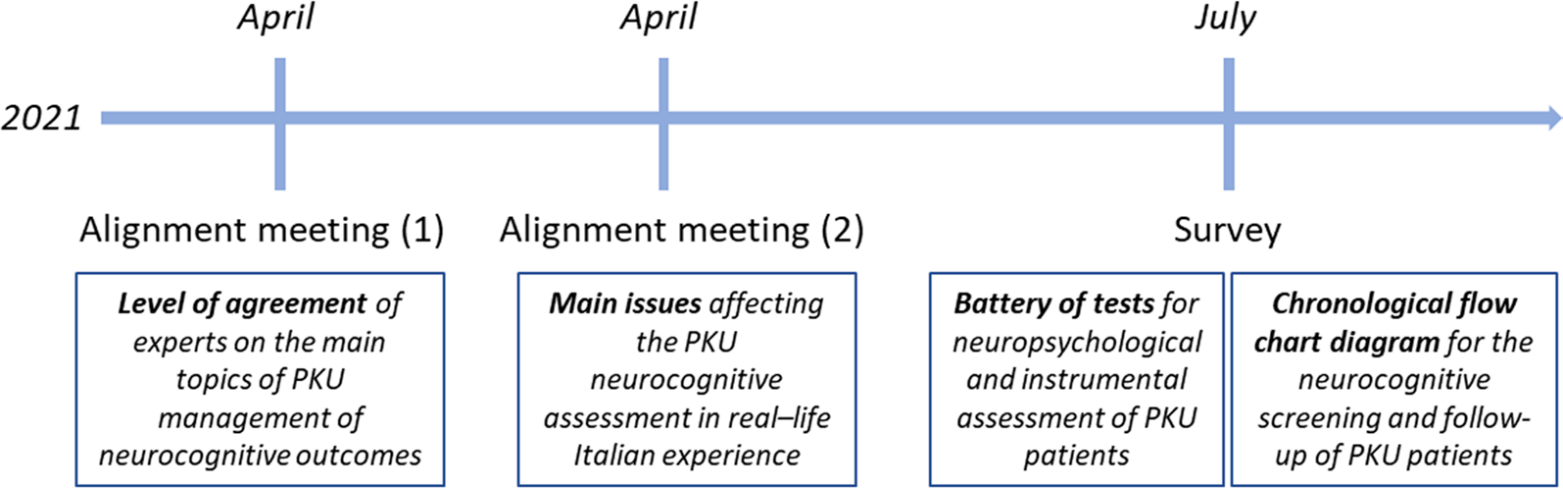
Program of the Expert meetings. The expert panel included six experts, two adult neurologists, three psychologists, and one child neuropsychiatrist. The first alignment meeting discussed the agreement concerning the main topics of PKU management; the second meeting defined the main issues affecting the neurocognitive assessment. The survey defined the set of tests and the flow chart for the neurocognitive assessment of the patients

The Expert Opinion was shaped through two alignment meetings and one survey (Fig. 1). The first alignment meeting discussed the results of one preliminary questionnaire administered to participants to assess their agreement/disagreement on the main topics of PKU management. The questionnaire consisted of seven questions with a voting possibility from 1 (total disagreement) to 9 (complete agreement). Marks ≥ 5 were considered in agreement, while ˂ 5 were in disagreement. Results were then extensively discussed with the help of a professional facilitator.

During the second alignment meeting, participants were interviewed about their setting and experience with the main issues affecting the neurocognitive assessment process in real-life PKU management.

The survey aimed to collect the recommendation of tools for neurocognitive and psychological follow-up of PKU patients in routine clinical evaluation.

Last, the experts were asked to define a chronological flow chart for PKU patients' cognitive, psychological, and neurological screening and follow-up.

## Results

### Level of agreement/disagreement of the experts on the principal topics of neurocognitive management of PKU patients

The participants discussed the results of the preliminary questionnaire in depth. The discussion focused mainly on questions correlating the frequency of neurological, neuropsychological, and psychiatric assessments with worsening school performance or comorbidities. At the end of the discussion, participants reached a high level of agreement on the neurocognitive assessment of PKU patients.

During the discussion, experts stated that the neurological assessment is to be performed at each visit. Visits are scheduled according to the age and needs of the patient. Neurological examinations should be performed repeatedly in children and adolescent, whereas, for adults, the guidelines suggest at least once a year in the absence of complications [3]. The frequency of the neurocognitive follow-up is increased according to the patient’s clinical and life conditions (causing clinical worsening/poor therapeutic compliance/comorbidity). IQ should be evaluated at 12 years and 18 years (as suggested by Burlina et al. [3]); a cognitive evaluation of pediatric patients could be necessary, for example, before starting the primary school. As already outlined by the national consensus document on PKU's management and pharmacological treatment, the multidisciplinary care team should include neuropsychiatrists, psychiatrists, psychologists, neurologists, metabolic pediatricians, and dietitians for the routine evaluation [3].

### Main issues affecting the cognitive, psychological, and neurological assessment process in real-life management of PKU patients

The main issues affecting the neurocognitive, psychopathological, and neurological assessment process in the daily clinical practice of the experts were assessed during the second session (Table 1).

**Table 1:** Main issues influencing the neurocognitive, psychopathological, and neurological assessment of PKU patients

Pediatric patient	Adolescent patient	Adult patient
Availability of a proper care setting	Availability of a proper care setting	Discontinuity of the doctor-patient relationship (inadequate periodicity of visits)
Patient availability	Patient availability	Lack of appropriate staff
Patient motivation for assessment	Patient motivation for assessment	Availability of patients to the assessment
Psychologist not always available at the time of the visit to the centers	Psychologist not always available at the time of the visit to the centers	Work commitments
Long evaluation times	Long evaluation times	Fear of the consequences of evaluations
Need to schedule several meetings	Need to schedule several meetings	Lack of risk perception
Patient's attentional time	Patient's attentional time	
Patient’s motivation for assessment	Patient’s motivation for assessment	
Difficulty by the patient to sustain the assessment	Difficulty by the patient to sustain the assessment	
Lack of parental awareness	Lack of parental awareness	
Fear of the family of the result	Fear of the family of the result	
Need to establish a good relationship with the family	Need to establish a good relationship with the patient	
	Need to establish a good relationship with the patient at a delicate time of transition to adulthood	

The panel agreed that while for pediatric and adolescent patients the main obstacles for adequate neurocognitive and psychopathological evaluation are logistical and operational, for adults there are factors related to acceptance of the medical condition, self-image, and the patient's perception/predisposition to risk.

### Tools for the cognitive, psychological, and neurological assessment of PKU patients according to their age

In choosing the examinations, the experts considered, on the one hand, the clinical significance and reliability of the test and, on the other, the feasibility of the examination in a context of daily clinical practice. The list was discussed and agreed by the experts.

Table 2 outlines the tests suggested for the clinical evaluation of PKU patients. Tests were subdivided for the pediatric, adolescent, and adult patients.

**Table 2:** Outcome measures in PKU patients

	Pediatric patient	Adolescent patient	Adult patient
*Outcome measures*			
IQ	Griffiths-III; WPPSI-IV; WISC-IV; LEITER-3 (in the case of language barriers and/or people with reduced/impaired verbal abilities)	WISC-IV; WAIS-IV; LEITER-3 (in the case of language barriers and/or people with reduced/impaired verbal abilities)	WAIS-IV; LEITER-3 (in the case of language barriers and/or people with reduced/impaired verbal abilities)
Executive Functions	BRIEF-P; BRIEF-2	BRIEF-2	BRIEF-Adult
*Assessment of neurocognitive domains*	In case of clinical worsening/poor compliance/comorbidity:		
Planning, mental flexibility and problem solving	WCST; TOL	WCST; TMT A-B; TOL; Semantic fluency	WCST; TMT A-B; TOL; Semantic fluency
Inhibitory control	Inhibition Test (NEPSY-II)	Inhibition Test (NEPSY-II)	SCWT
Visual-spatial memory	ROCF copy and memory	ROCF copy and memory; Corsi Test (Forward and Reverse)	ROCF copy and memory; Corsi Test (Forward and Reverse)
Visual-motor coordination	GPT; DSST; VMI	GPT; DSST; TMT A; VMI	GPT; DSST; TMT A
Short-term memory/Working memory	DGS (Forward and Reverse); Corsi Test (Forward and Reverse)	DGS (Forward and Reverse); Corsi Test (Forward and Reverse)	DGS (Forward and Reverse); Corsi Test (Forward and Reverse)
Processing speed	PSI (Wechsler scales); DSST	DSST; TMT A; PSI (Wechsler scales)	DSST; TMT A; PSI (Wechsler scales)
Sustained attention	Bells Test	TMT A-B	TMT A-B
Verbal fluency	Semantic fluency; Phonemic fluency	Semantic fluency; Phonemic fluency	Semantic fluency; Phonemic fluency
Verbal memory and learning	DGS (Forward and Reverse); Word List	RAVLT; Story Recall Test	RAVLT; Story Recall Test
Psychological classification (in case of clear emergence of specific symptoms)	Specialist counselling; Psychopathological status	Specialist counselling; Psychopathological status	Specialist counselling; Psychopathological status
Behavioral emotional assessment	CBCL 1 ½-5; CBCL 6-18	CBCL 6-18; YSR	ASR; BDI-II; STAI
Quality of life assessment	PKU-QoL	PKU-QoL	PKU-QoL
*Neurological evaluation*	Neurological examination	Neurological examination	Neurological examination
Tremor assessment	FTMCRST	FTMCRST	FTMCRST; TETRAS
*Instrumental Assessment*			
Brain MRI	Brain MRI in case of neurological signs	Brain MRI in case of neurological signs	Brain MRI in case of neurological signs

*WPPSI-IV* Wechsler Preschool and Primary Scale of Intelligence IV edition, *WISC-IV* Wechsler Intelligence Scale for Children IV edition, *WAIS-IV* Wechsler Adult Intelligence Scale IV edition, *BRIEF* Behavior Rating Inventory of Executive Function, *BRIEF-P* Behavior Rating Inventory of Executive Function–Preschool Version, *BRIEF-2* Behavior Rating Inventory of Executive Function–School Version, *BRIEF-A* Behavior Rating Inventory of Executive Function–Adult Version, *WCST* Wisconsin Card Sorting Test, *TMT* Trail Making Test, *TOL* Tower of London, *VMI* Visual-Motor Integration, *SCWT* Stroop Color and Word Test, *ROCF* Rey–Osterrieth complex figure test, *GPT* Grooved Pegboard Test, *DSST* Digit symbol substitution test, *DGS* Digit Span, *PSI* Processing Speed Index, *RAVLT* Rey Auditory Verbal Learning Test, *CBCL* Child Behaviour Checklist, *YSR* Youth Self Report, *ASR* Adult Self Report, *BDI* Beck Depression Inventory, *STAI* State-Trait Anxiety Inventory, *QoL* Quality of Life, *ASEBA* Achenbach System of Empirically Based Assessment, *FTMCRST* Fahn–Tolosa–Marin Clinical Rating Scale for Tremor, *TETRAS* Tremor Research Group Essential Tremor Rating Scale

The tests to assess the IQ selected by the panel of experts are universally recognized for the assessment of intellectual abilities and allow both clinical and diagnostic evaluation.

The rationale for the selection of the tests for the assessment of the CFx, was their good level of acceptance by patients and the specificity according to patient age. The experts selected tests which allows to evaluate specific skills. All selected tests are already widely used in the scientific literature for the analysis of other non-inherited metabolic diseases.

For the behavioral and emotional profile, experts chose the ASEBA scale (CBCL 1½-5yy; CBCL 6-18yy; YSR; ASR), one of the most widely used tool and the BDI and STAI scales.

For the QoL assessment, the panel of experts suggested the use PKU-QoL for all ages considered, as it was developed and validated specifically for patients with PKU [45].

The experts selected the age-specific tests according to five criteria: a) specificity by age, b) ability to detect dysfunctions, c) specificity for particular cases (e.g., foreign patients who do not have a good command of Italian), d) ease of administration, e) ability in detecting disease-related changes in QoL. As expected, no test met all five criteria; the main criterion for the choice of the individual task in each age group is listed below.

#### Pediatric patient

- test specificity by age: Griffiths-III, WPPSI-IV, WISC-IV, BRIEF-P, BRIEF-2, VMI, CBCL 1 ½-5, CBCL 6-18, Bells Test;
- ability to detect dysfunctions: WPPSI-IV, WSCT, TOL, ROCF copy and memory, DSST, VMI, DGS (Forward and Reverse), Bells Test, Semantic fluency, Phonemic fluency, Word List, CBCL 1 ½-5, CBCL 6-18;
- test specificity for particular cases: LEITER-3;
- ease of administration: BRIEF-P, BRIEF-2, DSST, DGS (Forward and Reverse), Bells Test, Semantic fluency, Phonemic fluency;
- specificity of the test in detecting disease-related changes: PKU-QoL.

#### Adolescent patient

- test specificity by age: WISC-IV, WAIS-IV, BRIEF-2, VMI, CBCL 6-18, YSR;
- ability to detect dysfunctions: WISC-IV, WAIS-IV, WSCT, TMT A-B, TOL, Semantic fluency, Phonemic fluency, ROCF copy and memory, Corsi Test (Forward and Reverse), DSST, TMT A, VMI, DGS (Forward and Reverse), RAVLT, Story Recall Test, CBCL 6-18, YSR;
- test specificity for particular cases: LEITER-3;
- ease of administration: BRIEF-2, TMT A-B, Semantic fluency, Phonemic fluency, DSST, TMT A, DGS (Forward and Reverse), Story Recall Test;
- specificity of the test in detecting disease-related changes: PKU-QoL.

#### Adult patient

- test specificity by age: WAIS-IV, BRIEF-Adult, ASEBA/ASR, BDI-II, STAI;
- ability to detect dysfunctions: WAIS-IV, WCST, TMT A-B, TOL, Semantic fluency, Phonemic fluency, SCWT, ROCF copy and memory, Corsi Test (Forward and Reverse), DSST, TMT-A, VMI, DGS (Forward and Reverse), RAVLT, Story Recall Test, ASEBA/YSR/ASR, BDI-II, STAI;
- test specificity for particular cases: LEITER-3;
- ease of administration: BRIEF-Adult, TMT A-B, Semantic fluency, Phonemic fluency, DSST, TMT-A, DGS (Forward and Reverse), Story Recall Test;
- specificity of the test in detecting disease-related changes: PKU-QoL.

#### Chronological flow chart diagram for cognitive, psychological, and neurological screening and follow-up of PKU patients

**Fig. 2:**
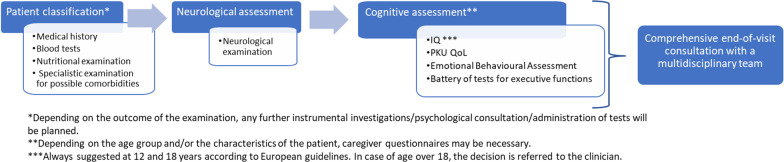
Flow-chart diagram for screening of PKU patients

**Fig. 3:**
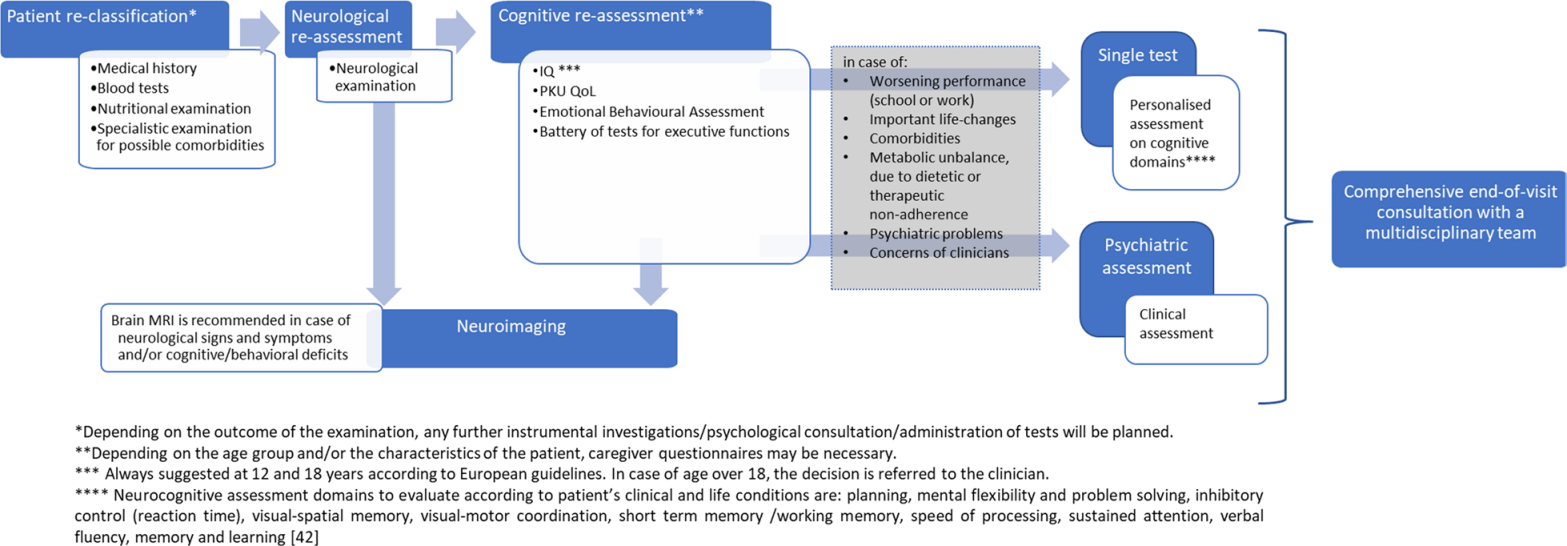
Flow-chart diagram for the follow-up of PKU patients

Figures 2 and 3 show a step-by-step chronological program proposed by the six experts involved in the study for the follow-up of PKU patients.

## Discussion

This paper intends to provide practical recommendations for the assessment of cognitive, psychological, and neurological outcomes in pediatric, adolescent, and adult patients with PKU. Since PKU is a disease characterized by subtle and heterogeneous neuropsychological and behavioral abnormalities, a specific set of cognitive tests should be implemented for PKU patients of different ages [27, 40]. The purpose of this paper was to reach an agreement on the selection of a panel of validated tests which could be administered to PKU patients from the pediatric age to adulthood. In the literature, only the PKU-QoL has been specifically developed to measure the QoL in PKU patients [46]. Therefore, for cognitive examination, we decided to have a different approach. We selected the most appropriate tests to analyze specific functions, which could be altered in PKU patients according to the impaired domains and corresponding to the age of the patients. Therefore, the major advantage of this panel of tests is that it focuses on specific domains, for a personalized approach to the PKU patient. We also considered the possibility to have the same test available in other local languages, to allow putative comparison of the analyzed cohorts of patient among different centers and countries.

For the neurological assessment, we strongly believe that a complete neurological examination should always be performed, especially for adult PKU patients and at least at the first visit.

We have identified two limitations in our work. First, the selection of a panel of tests has been necessarily done within a panel of tests developed for other medical settings rather than cognitive and psychological assessment given that no specific tests for PKU patients have been developed so far. However, the final choice was the result of the expert panelists who had previously applied these tests several times during their clinical practice with PKU patients. Second, our group involved only Italian centers, which may just reflect our clinical experience and approach to the PKU patient.

We hope this paper may stimulate the discussion on the most appropriate neuropsychological assessment of PKU patients, thus helping clinicians to better define the best clinical monitoring and improve their therapeutic management.

## Data Availability

Data sharing not applicable to this article as no datasets were produced or analyzed during the study.
