# Correction: Expert opinion of an Italian working group on the assessment of cognitive, psychological, and neurological outcomes in pediatric, adolescent, and adult patients with phenylketonuria

**DOI:** 10.1186/s13023-023-02644-2

**Published:** 2023-03-03

**Authors:** Filippo Manti, Stefania Caviglia, Chiara Cazzorla, Annamaria Dicintio, Andrea Pilotto, Alessandro P. Burlina

**Affiliations:** 1 Unit of Child Neurology and Psychiatry, Department of Human Neuroscience, University of Rome La Sapienza, Rome, Italy; 2 Unit of Clinical Psychology, Department of Neurosciences, Bambino Gesù Children’s Hospital, IRCCS, Rome, Italy; 3 Division of Inborn Metabolic Diseases, Department of Pediatrics, University Hospital, Padua, Italy; 4 Department of Metabolic Diseases and Clinical Genetics, Giovanni XXIII Children Hospital, Azienda Ospedaliero-Universitaria Consorziale Policlinico, 70126 Bari, Italy; 5 Neurology Unit, Department of Clinical and Experimental Sciences, University of Brescia, Brescia, Italy; 6 Neurology Unit, St. Bassiano Hospital, Bassano del Grappa, Italy

**Correction: Orphanet Journal of Rare Diseases (2022) 17:443** **https://doi.org/10.1186/s13023-022-02488-2**

Following publication of the original article [1], we have been notified that Table 2, column “Pediatric Patient” should be corrected as per below:

DGS (Forward and Reverse)

Also, reference 40 should be as follows:

40. Quinn J, Georgiadis A, Lewis HB, Jurecki E. Measuring burden of illness in phenylketonuria (PKU): development of the PKU symptom severity and impacts scale as a robust patient-reported outcome. Adv Ther. 2022;39:971–91.
